# A Patient With Regressed Diffuse Large B-Cell Lymphoma and Aggressive Follicular Lymphoma

**DOI:** 10.7759/cureus.15275

**Published:** 2021-05-27

**Authors:** Ashish K Sethi, Bushra Haq

**Affiliations:** 1 Medical Oncology, Allegheny Health Network, Pittsburgh, USA

**Keywords:** follicular lymphoma, r-chop therapy, hiv lymphoma, non-hodgkin lymphoma, diffuse large b-cell lymphoma, spontaneous regression

## Abstract

Diffuse large B‐cell lymphoma (DLBCL) and follicular lymphoma (FL) are the two most aggressive forms of non‐Hodgkin lymphomas (NHLs). Spontaneous remission of DLBCL is a rare phenomenon. Immune system activation has been observed to play a significant role in the regression of untreated disease on some occasions. We present a case of DLBCL in a 75-year-old male patient who has been free of disease for two months without any treatment due to possible immune-related mechanism, but later he presented with FL.

## Introduction

Lymphomas are the malignancies of the lymphoid system that can arise from B lymphocytes or T lymphocytes or natural killer (NK) cells depending on their stages of maturation. They are categorized into Hodgkin lymphoma and non-lymphoma Hodgkin (NHL). Diffuse large B-cell lymphoma (DLBCL) and follicular lymphoma (FL) are the most common forms of NHLs. There are roughly 150,000 new cases of DLBCL diagnosed globally each year [1]. The disease is seen frequently in whites followed by African Americans and Asians, with a male preponderance. The overall incidence of NHL or DLBCL increases exponentially with age.

DLBCL commonly presents as lymphadenopathy or rapidly growing mass with B symptoms, which include fever, night sweats, and weight loss. Extranodal sites are seen in approximately 40% of patients with DLBCL [2]. The most common sites of extranodal spread of disease are the gastrointestinal tract, skin, bones, head and neck, central nervous system, skeletal system, and testicles [3]. Due to the hematogenous spread, DLBCL can remain asymptomatic until a late disease stage, and symptoms mainly depend on the site of involvement.

We present an unusual case of a 75-year-old male patient diagnosed with DLBCL harboring poor prognostic features of MYC and BCL2 protein coexpression showing spontaneous regression of lymphadenopathy without any intervention or treatment and later presenting with FL.

## Case presentation

A 75-year-old Caucasian male was diagnosed with DLBCL based on clinical and diagnostic evaluation. Prior to his confirmed diagnosis, on routine physical evaluation at an outpatient setting he was observed to have an axillary lymphadenopathy that was associated with right-sided hilar adenopathy on chest X-ray. The patient denied having complaints of weight loss, fever, chills, night sweats, shortness of breath, or chest pain in the past. His medical history included type 2 diabetes mellitus, hyperlipidemia, aortic stenosis, and coronary artery disease (CAD). The patient also underwent transcatheter aortic valve replacement (TAVR) and coronary artery bypass grafting (CABG) for his aortic stenosis and CAD.

**Figure 1:**
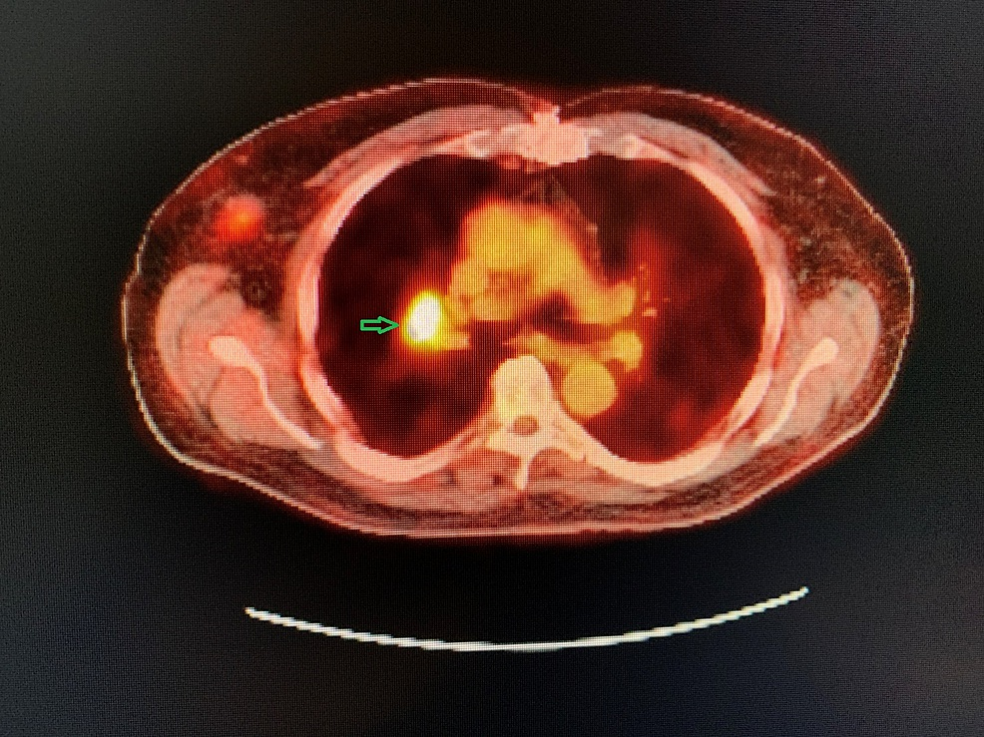
PET scan showing right hilar mass (green arrow)

**Figure 2:**
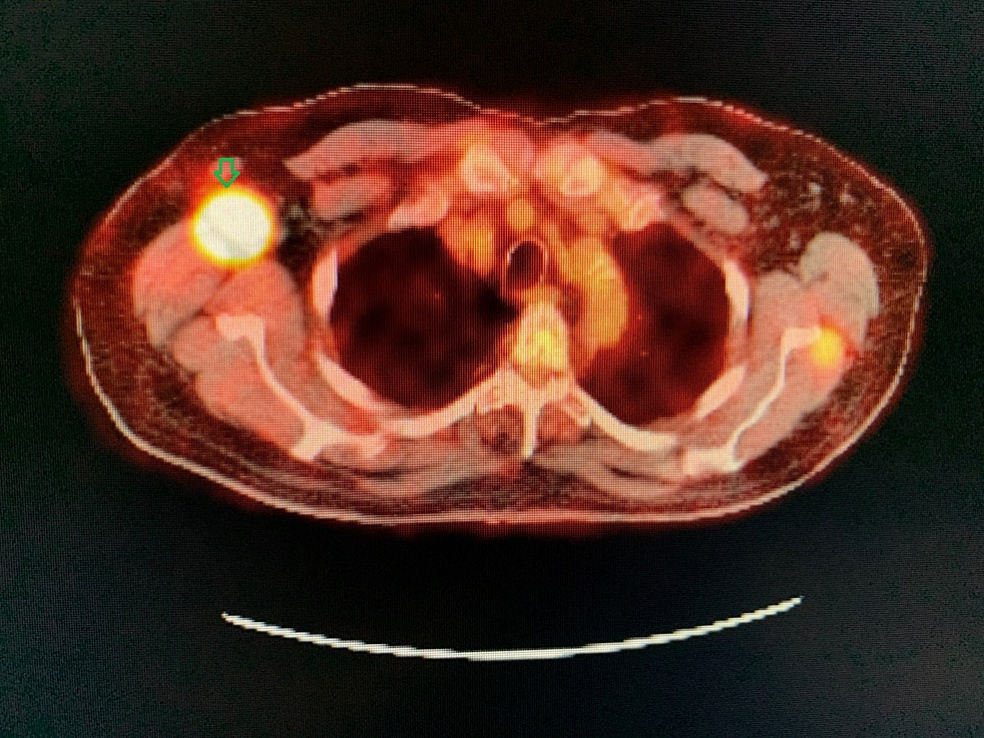
PET scan showing right axillary mass (green arrow)

**Figure 3:**
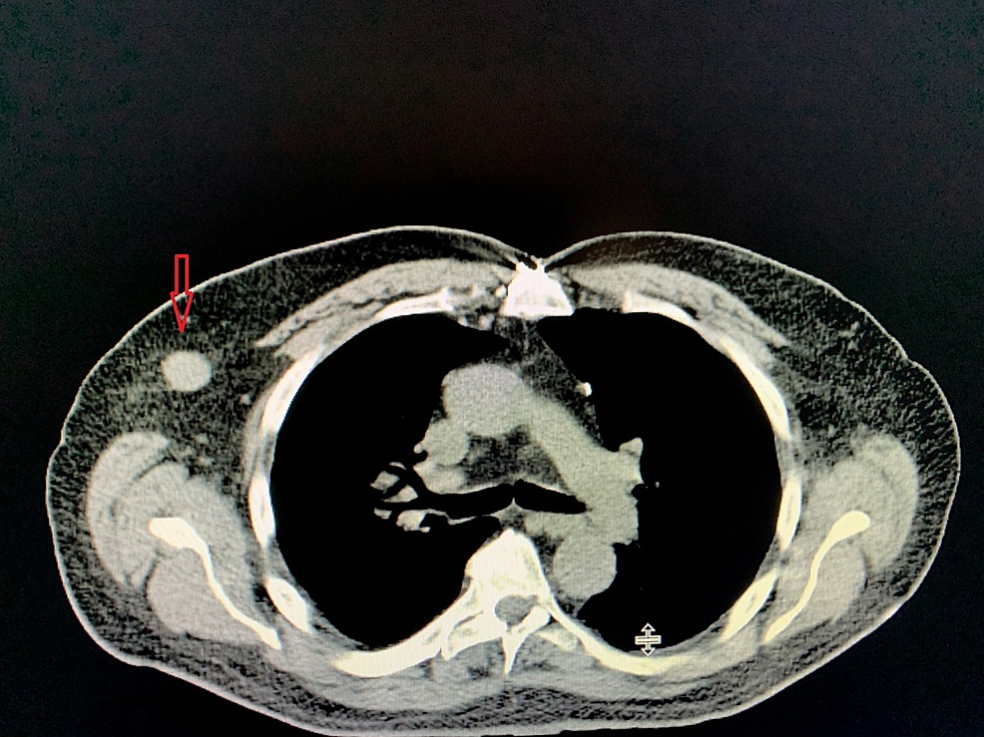
CT scan showing axillary mass/lymphadenopathy (red arrow)

**Figure 4:**
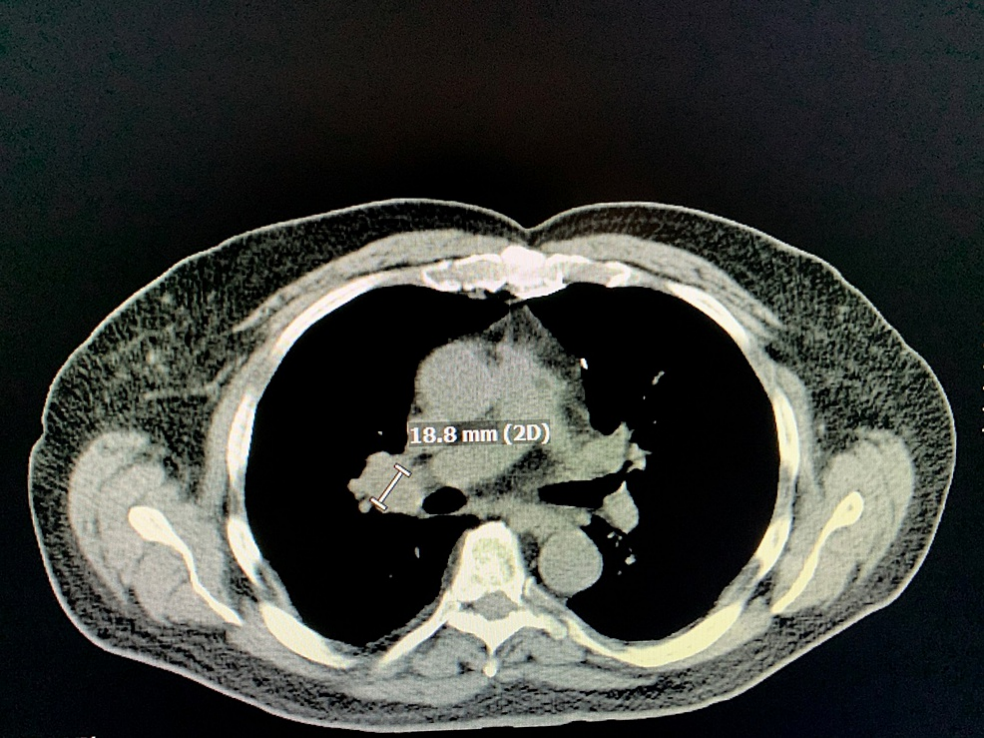
CT scan showing right hilar mass

**Figure 5:**
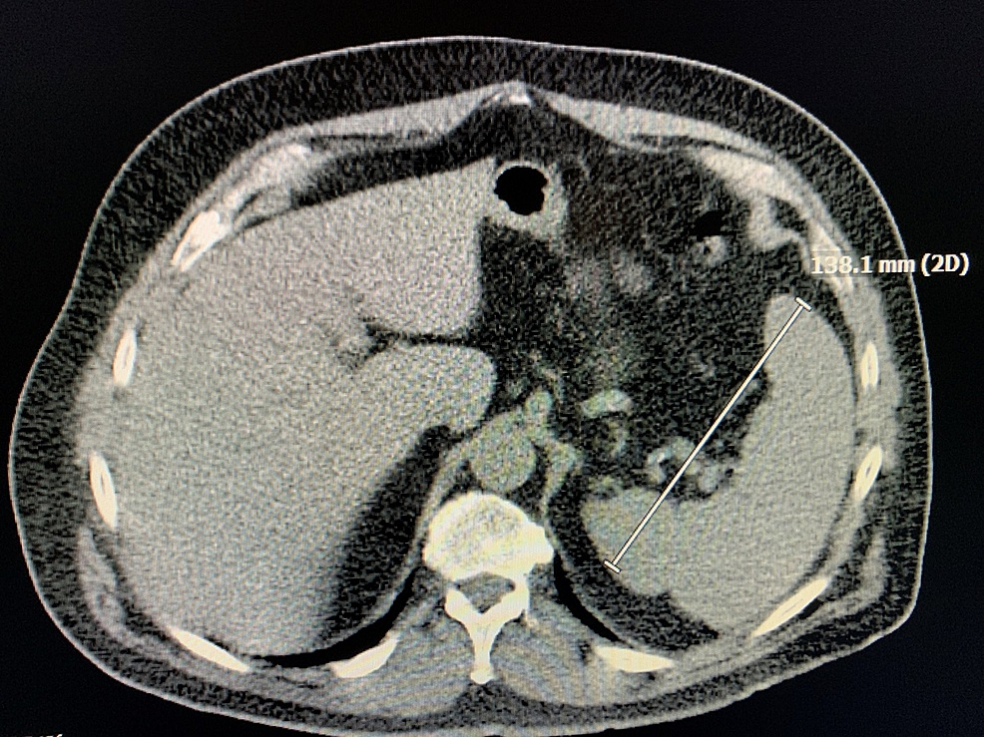
CT scan showing splenomegaly

During the evaluation for DLBCL, the patient underwent positron emission tomography (PET) scan, which revealed an enlarged and increased standardized uptake value (SUV) in the right hilar lymph node (Figure 1), right axillary lymph node (Figure 2), inter-aortocaval lymph node, and retroperitoneal lymph nodes. Similar enlargement of axillary node and hilar nodes with splenomegaly was observed on computed tomography (CT) scan (Figures 3-5).

**Figure 6:**
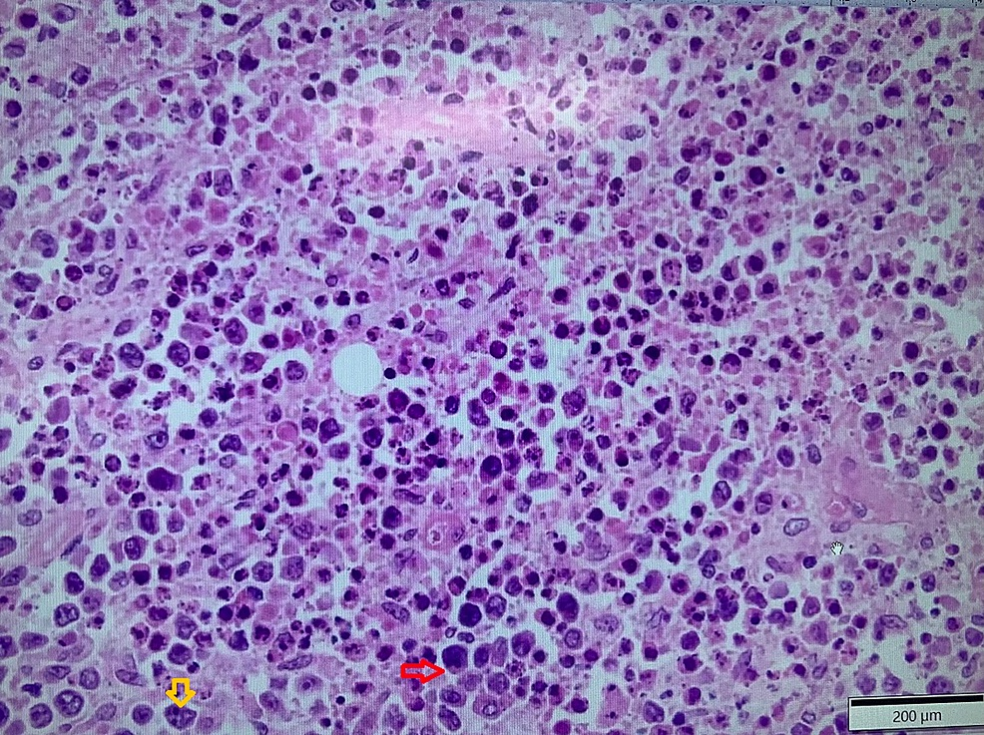
Axillary lymph node biopsy Large lymphocytes (red arrow), nuclear pleomorphism, and prominent nucleoli (yellow arrow) can be seen.

Excisional biopsy of the right axillary mass was performed, which showed features of DLBCL (Figure 6). Furthermore, flow cytometry demonstrated a cluster of differentiation (CD) 5 negative and CD10 negative clonal B-cell population with lambda light chain restriction confirming B-cell lymphoproliferative disorder. Right upper lobe fine needle aspiration cytology (FNAC) was negative for malignancy. Fluorescence in situ hybridization (FISH) analysis revealed abnormal cell line positive for BCL6 and negative for MYC and BCL2 rearrangements.

**Figure 7:**
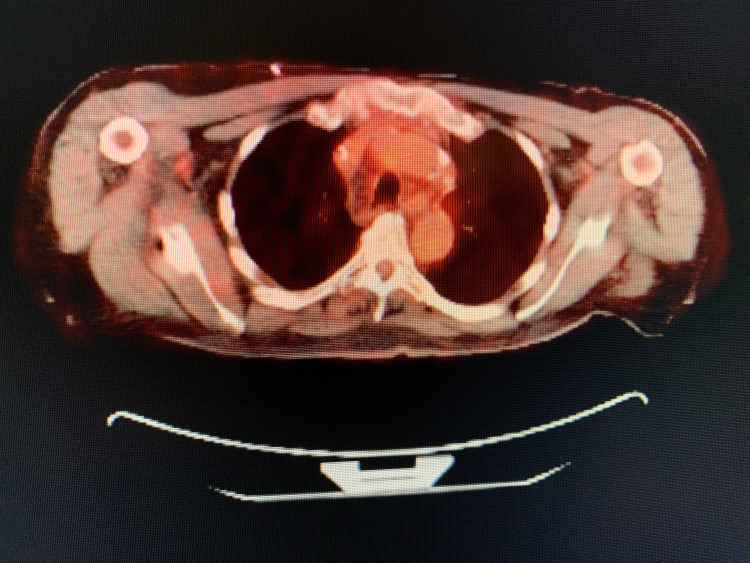
PET scan showing no axillary mass

**Figure 8:**
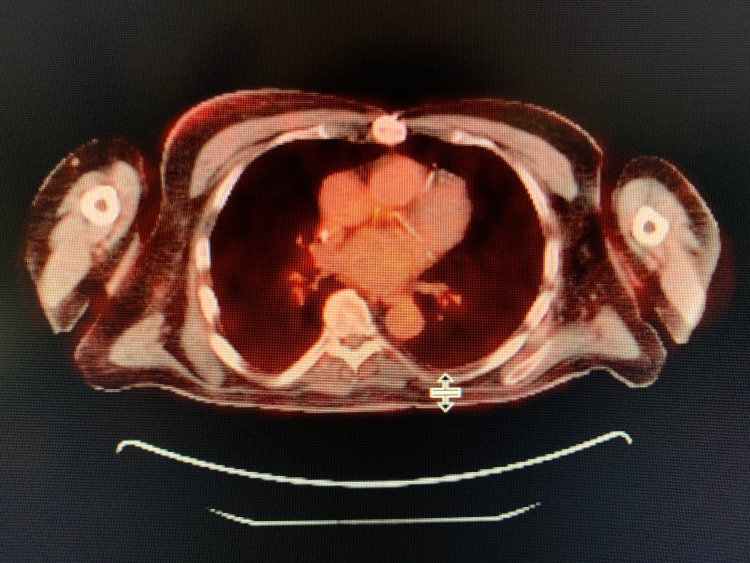
PET scan showing no SUV/FDG uptake in the hilar region SUV, standardized uptake value; FDG, fluorodeoxyglucose

Interval resolution of previously seen hypermetabolic right axillary node (Figure 7), right hilar node (Figure 8), and abdominal lymphadenopathy was noticed on its own on PET scan after two months from the date of diagnosis without any form of treatment, which was observed to be an unusual phenomenon.

**Figure 9:**
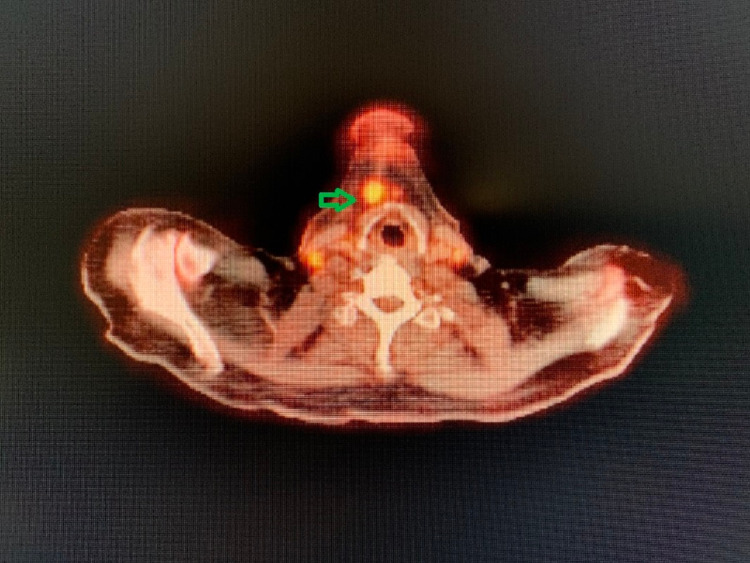
PET scan showing submental lymhadenopathy FDG uptake is seen in the right submental lymph node FDG, fluorodeoxyglucose

Later, on follow-up PET scan, multiple new foci of increased SUV in the right submental lymph node (Figure 9), spleen, and left femoral shaft were observed.

**Figure 10:**
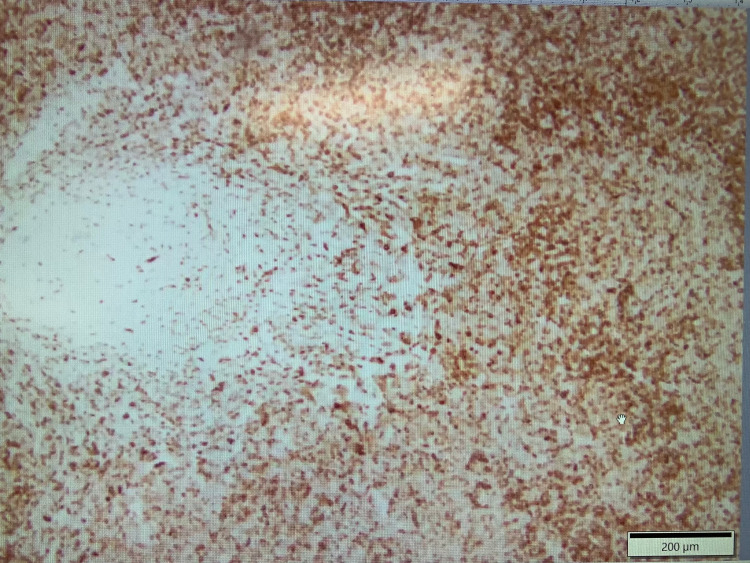
Submental biopsy showing follicular lymphoma (CD21 positive)

**Figure 11:**
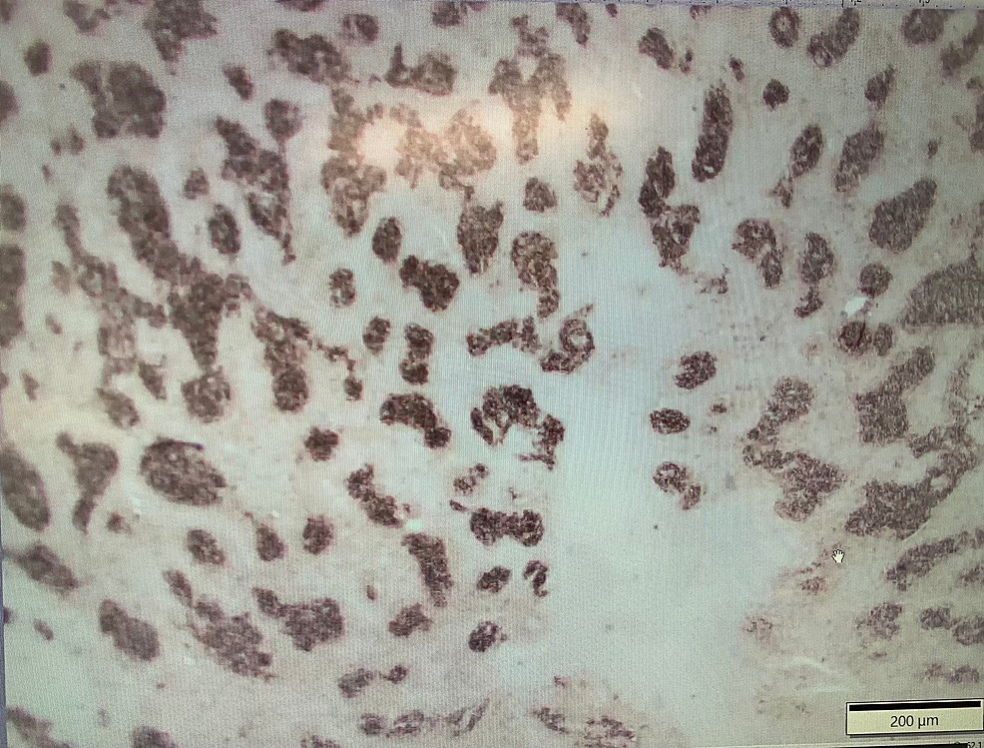
Submental lymph node biopsy showing follicular lymphoma (CD21 positive with dendritic meshwork pattern)

Subsequently, excisional biopsy of the submental lymph node confirmed CD21 positive (Figures 10, 11) and BCL2 negative stage 3B FL. Rituximab, cyclophosphamide, doxorubicin, vincristine, and prednisone (R-CHOP) therapy is now planned for the patient based on his complaints of persistent fatigue and newly diagnosed aggressive FL. The patient's lactate dehydrogenase (LDH) levels were also persistently elevated on subsequent disease monitoring.

## Discussion

The Ann Arbor staging system (Table 1) illustrates the extent of disease, age, presence of extranodal involvement, elevated LDH, and performance status based on the international prognostic index (IPI), which are all prognostic factors in overall survival in B-cell NHL [4].

**Table 1:** Ann Arbor staging system: lymphoma

Stage	Clinical manifestation
I	Single lymph node or single extra-lymphatic organ
II	>2 lymph node regions on the same side of the diaphragm
III	Both sides of the diaphragm involved; may have spleen or local tissue involvement
IV	Multiple/disseminated foci involved with >1 extra-lymphatic organs (i.e., bone marrow)

The standard first-line chemotherapy treatment of DLBCL is the combination of R-CHOP. Although DLBCL can be cured with first-line chemotherapy in more than half of all patients, up to 50% of patients do not respond to initial treatment or relapse after showing preliminary response [5]. Spontaneous regression of lymph nodes and remission of disease in aggressive lymphomas such as DLBCL and FL is a rare phenomenon, and data are limited in the medical literature. The physiology of spontaneous remission of the disease remains unknown. Regression is more commonly observed with groups of tumors such as embryonal tumors in children, carcinoma of the breast, adenocarcinoma of the kidney, neuroblastoma, malignant melanoma, sarcomas, and carcinoma of the bladder and skin [6]. Armstrong et al. hypothesized that immune reconstitution inflammatory syndrome following the initiation of antiretroviral therapy has played a role in the regression of the human immunodeficiency virus (HIV)‐associated plasmablastic lymphoma of the oral cavity [7]. The phenomenon of regression in lymphoma has also been postulated to be immune‐mediated secondary to activation from viruses, such as Epstein‐Barr virus, or local trauma from the biopsy site [7-9].

## Conclusions

The patient in our case opted for surveillance and showed no evidence of disease recurrence at two months since the biopsy results of DLBCL. The remission occurred via an unknown mechanism, possibly related to immune system activation. However, FL at a different site with a history of DLBCL in remission in the same patient is also an uncommon phenomenon. Thus, this case provides further insight into the role of immune system activation in regression of aggressive lymphomas such as DLBCLs or FLs.
